# Enhancing healthcare accessibility measurements using GIS: A case study in Seoul, Korea

**DOI:** 10.1371/journal.pone.0193013

**Published:** 2018-02-20

**Authors:** Yeeun Kim, Young-Ji Byon, Hwasoo Yeo

**Affiliations:** 1 Department of Civil and Environmental Engineering, Korea Advanced Institute of Science and Technology, Daejeon, Republic of Korea; 2 Department of Civil Infrastructure and Environmental Engineering, Khalifa University, Abu Dhabi, United Arab Emirates; University of Saskatchewan, CANADA

## Abstract

With recent aging demographic trends, the needs for enhancing geo-spatial analysis capabilities and monitoring the status of accessibilities of its citizens with healthcare services have increased. The accessibility to healthcare is determined not only by geographic distances to service locations, but also includes travel time, available modes of transportation, and departure time. Having access to the latest and accurate information regarding the healthcare accessibility allows the municipal government to plan for improvements, including expansion of healthcare infrastructure, effective labor distribution, alternative healthcare options for the regions with low accessibilities, and redesigning the public transportation routes and schedules. This paper proposes a new method named, Seoul Enhanced 2-Step Floating Catchment Area (SE2SFCA), which is customized for the city of Seoul, where population density is higher and the average distance between healthcare-service locations tends to be shorter than the typical North American or European cities. The proposed method of SE2SFCA is found to be realistic and effective in determining the weak accessibility regions. It resolves the over-estimation issues of the past, arising from the assignment of high healthcare accessibility for the regions with large hospitals and high density of population and hospitals.

## Introduction

Accessibility to medical services is one of the most critical measures in determining the quality of life [1]. From the perspective of social equity, everyone should have the opportunity to access such services equally. However, it is a challenge to achieve such equity with various obstacles such as economic and geographical issues.

In the late 1980s, the Republic of Korea started a national healthcare insurance program for all citizens and has established medical infrastructures throughout the nation by paying attention to different attributes including race, age, income, and residential/commercial/industrial activities. Achieving the equity in medical services has been one of the major priorities in making health administrative decisions in Korea. Thus, the current status of medical accessibility distributions in major cities including its capital, Seoul, have been studied.

Seoul City has a population of approximately 10 million, which is about one-fifth of the national population. The surrounding regions of Seoul, known as the Greater Seoul Area (GSA), accounts for an additional 30% of the national population [2]. Resolving the medical accessibility issues in Seoul and the GSA alone account for the half of the national population of Korea. Therefore, Seoul is the best venue to conduct a medical equity study for potential improvements that can later be extended to other regions of Korea.

Internationally, there are many active researches on the topic. In general, there are multiple approaches in assessing the healthcare accessibility. The simplest way is to use the ratio between the supply and demand within a standard area [3]. However, a major weakness of this method is that there are no considerations given on distances within the area. The distance-to-nearest-primary-care-physician method matches the demand point to the nearest supply point. It is noted that this method is not effective in a metropolis where many alternatives exist [4]. On the other hand, the average method uses the mean of distances to all healthcare supply points from a patient’s location. Although it provides a simple way to find the optimal location, this method has a crucial limitation as it overestimates the influence of healthcare supplies near the outskirts of the city. Moreover, the gravity method measures the accessibility of healthcare supplies considering their service availability within acceptable critical boundary distances [5]. Meanwhile, the floating catchment area (FCA) method or other methods using substitutions of functions of travel times for distant coefficients have been suggested to solve the limitations such as the unintuitive results and vague standards used for determining the distant coefficient [6]. An additional problem with the methods mentioned above is that the impact of neighboring areas is not taken into account sufficiently. To overcome this, 2 step floating catchment area, which integrates an interaction between demand and supply with demand-to-supply ratio, has emerged.

Luo and Wang developed 2SFCA method, merely a special case of the gravity method, in efforts to enhance the previous FCA approach for determining accessibility to healthcare measures [7]. McLafferty further considers the available transportation modes, types of medical services, and the healthcare demands [8]. Other researchers have started considering both spatial and non-spatial factors [9–12]. The 2SFCA method has a drawback that all hospitals within the same critical distance boundary are equally considered. In order to overcome this limitation, other researchers developed enhanced versions of the 2SFCA method. Enhanced 2SFCA (E2SFCA) first divides the distances in sections and penalizes further-distance sections [13]. Kernel Density 2SFCA (KD2SFCA) uses a Gaussian function in determining the penalties or costs [14]. More recently, Bauer and Groneberg proposed integrated FCA method (iFCA) by using logistic cumulative distribution function, which employs median and standard deviation of all population-to-physician distances as parameters, instead of the commonly used Gaussian function [15]. There are more complex methods that recognize the issue of over-estimating accessibility when there are multiple hospitals in a dense region. In reality, patients have different preferences and there are competitions among the hospitals. 3SFCA considers the competition using the user’s selection-weight and modified 2SFCA (M2SFCA) factors for computing the absolute and relative distances [16, 17]. Despite the improvement of these methods, they do not take into consideration the socio-demographic factors. Some researchers have suggested a method that takes into account multiple travel modes, which suggests a partial solution to this problem by considering the information on the car ownership [18, 19]. In addition, the family of methodologies has suffered from another limitation in that all physicians and population use fixed catchment sizes. Luo and Whippo attempted to solve this problem by dynamically determining the catchment size to satisfy a certain physician-to-population ratio [20]. Another study related to this issue has used dynamic catchment sizes considering the remoteness level of the area [21].

In Korea, 2SFCA method and its extended versions, E2SFCA and KD2SFCA, have been used in the past. However, these methods overestimate the accessibility to healthcare as the number of hospitals in the vicinity increases. The study cited in the previous studies are mostly located in the U.S. [7, 10–14,16,17]. These cities have different characteristics from the Korean cities. The U.S. has a larger geographical area and a lower hospital density than Korea. By 2015, the number of registered hospitals by country was 5,564 in the U.S, and 1,937 in Korea, but the hospital density was 0.00057 and 0.019 hospitals/km^2^, respectively. The Korean hospital density is approximately 30 times bigger than the US [22, 23]. Seoul has a higher hospital density (0.047), and it gets even higher when small-size hospitals without beds are additionally considered (13.68). Accordingly, the over-estimation of accessibility can be more significant in cities in Korea compared to the regions that have been studied so far. Especially in Seoul, where hospitals of various sizes are mixed, the serviceable range of large hospitals, including mega-complexes, should be considered differently. There are many large hospitals including mega-complexes and their serviceable range should be differently considered.

Geographically, Seoul is a relatively small city and its geographical size is 37 km by 30 km with an area of 605.27 km^2^ and high population density. In addition to the high population density, the income level, as well as car ownership vary within the city. The income differential indices (IDI) represent a number rated from 1 to 10 where a larger number is associated with a higher income. In 2012, the highest and lowest municipal blocks (MBs) in Korea are found to be 9.62 and 2.24 respectively; similarly, the number of cars owned per household is 3.92 and 0.14 respectively [2]. The geographic area of Seoul is only 0.6% of the nation, yet it contains many different economic classes, which leads to an unequal distribution of quality of life including accessibility to medical services. Often, the lower income population turns out to receive the penalties arising from the inequity. The low income population tends to depend on the public health care services more than the privatized ones.

Therefore, the previous methods and approaches for determining healthcare accessibility should not be directly applied to Seoul. There is a need to develop a customized new method that measures the healthcare service accessibility for Seoul and other international cities with similar conditions. Accordingly, this paper proposes a new healthcare accessibility measurement method for Seoul named, Seoul Enhanced 2-Step Floating Catchment Area (SE2SFCA) method.

## Methods

### 1. Healthcare accessibility measurement method for Seoul

In this section, we present a brief explanation on the 2SFCA and E2SFCA, which are the underlying models of SE2SFCA. Then, the proposed SE2SFCA (Seoul enhanced 2 step floating catchment area) method is formulated from the extension of the existing methods.

#### 1) 2SFCA (2 Step Floating Catchment Area)

The 2SFCA is one of the most popular methods to measure healthcare accessibility. This method considers the catchment area for both population and healthcare service and consists of two steps in general. In the first step, the supply to demand ratio (R_j_) is calculated at each location of a healthcare facility (j) within the critical travel time (t_0_) boundary. It is calculated by dividing the number of supply (S_j_) by the total population located at k within the critical travel time (t_0_). S_j_ depends on the number of healthcare employees or the number of beds.

$$R_{j}=\frac{S_{j}}{\sum_{t_{kj}\in t_{0}}P_{k}}$$ (1)

In the second step, the accessibility to healthcare (A_i_) and opportunities for healthcare per person are calculated as a sum of the supply to demand ratio R_j_ for all facilities falling within the critical travel time from each population (i).

$$A_{i}=\sum_{j\in (t_{ij}\leq t_{0})}R_{j}=\sum_{j\in (t_{ij}\leq t_{0})}\frac{S_{j}}{\sum_{k\in (t_{kj}\leq t_{0})}P_{k}}$$ (2)

#### 2) E2SFCA (Enhanced 2 Step Floating Catchment Area)

In the 2SFCA method, the accessibility is assumed equal among all locations within the critical distance boundary, while the locations outside the boundary are unreachable at all. In order to overcome this drawback, E2FCA penalizes the regions that are further away even if they are within the boundary [24]. If the supply location is further away from the demand location, there is a lower chance that this particular healthcare service is selected for use. Similar to 2SFCA, E2SFCA consists of two steps and in each step, the area within the critical distance is gradually penalized by distance.

Critical boundary is divided into several distance rings (T_r_) with different weights (W_r_) to penalize further distances. Weight values which are assigned to different rings are measured by a distance decay function. Generally, Gaussian, inverse power, and Exponential functions are used for distance decay functions [25]. Two steps of E2SFCA are expressed as below:

$$R_{j}=\frac{S_{j}}{\sum_{k\in (t_{k}\leq T_{r})}P_{k}W_{r}}$$ (3)

$$A_{i}=\sum_{j\in (t_{ij}\leq T_{r})}R_{j}W_{r}$$ (4)

#### 3) SE2SFCA (Seoul Enhanced 2 Step Floating Catchment Area)

However, E2SFCA has few limitations. The critical distance is determined regardless of the size of hospitals. There are more than 30 large hospital complexes in Seoul and some of them have more than 500 healthcare employees. Larger hospitals can provide services with larger area coverage. With this in mind, in this study, the critical travel time t_0_ for the first step is modeled as a function of the number of physicians (S_j_) in the first phase of E2SFCA.

$$t_{0}=\left\{ \begin{array}{cc} 30e^{{-(\frac{S_{j}-S_{1}}{S_{1}})}^{2}}\;min & \left(S_{j}\leq S_{1}\right)\\ 1\;hr & \left(S_{1}<S_{j}\leq S_{2}\right)\\ 2\;hr & \left(S_{2}<S_{j}\right) \end{array}\right.$$ (5)

Where S_1_ and S_2_ are the standard number of physicians for distinguishing healthcare facilities between a regular hospital, hospital complex and large hospital complex. In Korea, a healthcare facility is categorized into regular hospital, hospital complex and large hospital complex in accordance with the size and the number of provided medical specialties. In this paper, the size criteria for dividing the three categories are 58 and 440 respectively according to Korea Health Industry Development Institute. Compared to regular hospitals, hospital complexes have fewer impedances on travel time because they differ significantly in terms of the number of diseases that they can handle. Considering this, the critical travel time for hospital complex and large hospital complex are set to 1 and 2 hours, respectively. On the other hand, under the assumption that equal quality of medical services is provided, a service range of regular hospital is set to be proportional to its size. A critical boundary of a regular hospital is derived by a Gaussian distribution. It is calculated by subtracting S_1_ from S_j_ and dividing it by a constant number (S_1_) to satisfy the maximum of critical travel time and the value of 30 minutes is generally used in the previous researches [7, 13]. The t_0_ for hospital complex and large hospital complex are arbitrarily set.

Although the probability of a person approaching a certain healthcare facility is affected by many factors such as income level and car possession, E2SFCA assumes that each person has a same approaching possibility. To solve this limit, a critical distance boundary (D_t_) calculated from the critical travel time, is modeled as a function considering the travel mode of each population.

$$D_{t}=\{\min\left(1,c_{i}\right)\times v_{c}\times t_{0}\}+\{(1-\min\left(1,c_{i}\right))\times v_{p}\times (t_{0}-t_{w})\}$$ (6)

Where c_i_ is the number of private vehicles per person at population location i, and v_c_ and v_p_ are average speeds of private vehicle and public transportation modes, respectively. In the case of using the public transportation, the travel time boundary is also penalized by subtracting the waiting time (t_w_) from t_0_. In order to identify the hospitals included in the catchment area for each population, travel time is converted to travel distance, and the circle using this travel distance as a radius is selected as a catchment area. According to this formula, for a population that owns more than one private vehicle, the critical distance is calculated as the product of the private vehicle speed and the critical travel time. In case of less than one vehicle, the proportion of using public transportation is increased in inverse proportion to the status of vehicle ownership. As the public transportation penalize the travel time boundary, the population with low vehicle possession is generally constrained to travel. These constraints reflect the accessibilities of different socioeconomic status.

**Fig 1:**
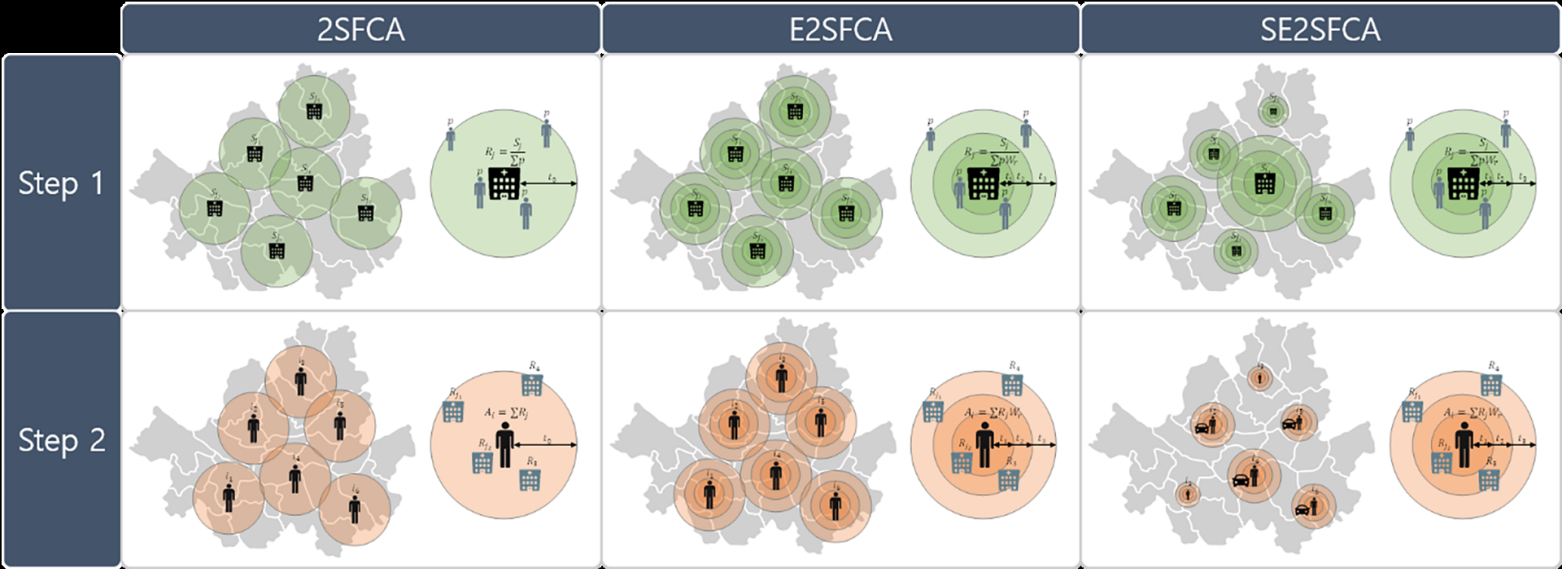
Comparison of three methods for healthcare accessibility measurements.

Fig 1 shows the concept of the suggested method of SE2SFCA compared with others. E2SFCA follows the most basic 2SFCA model, but solves the problem of having consistent access within the catchment area by applying a discrete distance-decay function. The proposed method evolved by adjusting the catchment size in different ways in two separate steps. In the first step, the service range of the hospital is defined by applying different catchment sizes according to the hospital sizes under the assumption that their attractiveness depends on the size. In the second step, multiple travel modes are considered. The critical travel time is the same for all populations, but the travel distance varies according to the travel mode.

### 2. Accessibility measurement for private and public healthcare facilities

The accessibility measurements are applied differently to private and public healthcare facilities considering their unique characteristics. QGIS software is used for the spatial analysis, including combining several attributes by location, calculation of metric and visually illustrating the results of the analysis for intuitive comparisons among the different districts of Seoul.

#### 1) Accessibility measurement for private healthcare facilities

In order to measure the accessibility for private hospitals, a new approach, SE2SFCA (Seoul 2 step floating catchment area) model is proposed in this paper. This paper uses the number of healthcare employees for Sj since it is generally considered as a more crucial factor than the number of beds in the most cases of Seoul, which has thousands of hospitals without beds [2]. Critical travel time boundaries are categorized into three sub-regions based on the travel time in cars; one-third of t_0_, two-third of t_0_, and t_0_. The weight of each case is found to be 1.00, 0.68, 0.22 respectively from the Gaussian decay function, which takes the form ${f(t}_{0})=e^{-\frac{{\left(2t_{0}\right)}^{2}}{1000}}$. In the second step, t_0_ is set at 15 minutes for all populations since there is no preference for a particular hospital with the assumption that all hospitals provide the same quality of service for the same disease.

The vehicle speed data is provided in Seoul Statistics to estimate the travel distance [2]. The average speed of a private vehicle is differently applied depending on the district considering the regional traffic. In case of the public transportation, the same speed is used throughout Seoul due to the data limitation. The waiting time of the public transportation is obtained through the household travel survey data and it is applied differently in the MBs [26].

#### 2) Accessibility measurement for public healthcare facilities

The accessibility assessment for public hospitals is significantly different. The public hospitals are different from the private hospitals in terms of size and providing services. The public hospitals have similar sizes and focus on primary services as they are built for a specific purpose of serving the public. Unlike privatized ones, the hospital density is low. In each district, there are generally two public hospitals in Seoul.

Since the number of physicians in public healthcare facilities is generally very small such as one or two in each facility, if we use Eq (5), the catchment size becomes too small. Therefore, for the public hospital, the first line of Eq (5) is applied by using different standard S_p_ instead of S_1_ as below.

$$t_{0}=30e^{-{\left(\frac{S_{j}-S_{p}}{S_{p}}\right)}^{2}}\;min$$ (7)

Where S_p_ is the standard number of physicians in public healthcare facility, which is set to two. According to this formula, the critical travel time t_0_ is set to be longer than that of private hospitals [27].

In addition, as one of the main reasons of selecting a public hospital over a private one is the cost, this paper assumes that the top 10% income group do not use the public hospitals. Therefore, the rest of the populations are used for assessing the accessibility to public hospitals.

#### 3) Data requirements for calculation

For the study, data from the City of Seoul is used for the accessibility analysis. The city consists of about 10 million population, 24 districts and 423 municipal blocks (MBs). This paper first conducts an analysis by aggregating on a district level throughout the city, then, further applies the MB level analysis for the districts with unique and interesting results. Table 1 shows the data used for the analysis.

**Table 1:** Data reference.

Reference	Data
Seoul Metropolitan Government Series (2005 ~ 2014)	Household travel survey data (2010) Resident population (2005) Estimated income distribution (2005) Vehicle registration (2012) Vehicle passing speed (2014) Hospital counts (2005) Healthcare employees (2012) Public healthcare facilities (2014) Healthcare employees for public facilities (2014)

The residential population (2005), estimated income distribution (2005), and hospital counts (2005) are presented in a 100m-by-100m cell format and the center point of each cell contains the information such as total population, average estimated income level, and the number of physicians. The population data from 2005 to 2014 are corrected by multiplying the population growth rate of 1.14 every year. The hospitals used for the analysis of this study are both public and private general hospitals, clinics, and doctor’s offices excluding special clinics such as the gynecologist, dentist, and Chinese traditional medicine.

The City of Seoul provides results from their public commuting trend analysis study, Seoul Metropolitan Government (2005~2014). The statistics office of the city provides the list of healthcare facilities (by MBs), hospital employees (by MBs), public clinics (by MBs), employees of public clinics, vehicle registrations and ownership (by MBs), and traffic speeds by districts for each day of the week. The data is used to estimate the approachability to healthcare employees. For the estimation of passenger waiting times, the scheduling information on public transit services is used. For the healthcare-employee counts, residing doctors are considered excluding their assistants, nurses, and administrative staffs. For the vehicle registration data, only the private vehicles are counted excluding governmental and industrial/commercial vehicles.

## Results

### 1. Accessibility to private healthcare facilities

The population grid illustrates the population of the City of Seoul and 10,369,593 people are analysed. Within the 3,647 hospital grids, there are 7,741 large and regular hospital complexes, private hospitals, and clinics with 26,236 doctors.

The above results in 2.53 doctors per 1,000 people in Seoul. Table 2 compares the density of healthcare staffs in Korea, OECD, and other countries and it shows that Korea is relatively high ranked in healthcare manpower.

**Table 2:** Healthcare manpower per 1000 residents [28].

	Seoul	Rep. of KOREA	OECD	Other countries
Number of physicians/1000	2.53	2.08	2.78	1.54

Reference: Healthcare manpower per 1000 residents (WHO, 2015)

**Fig 2:**
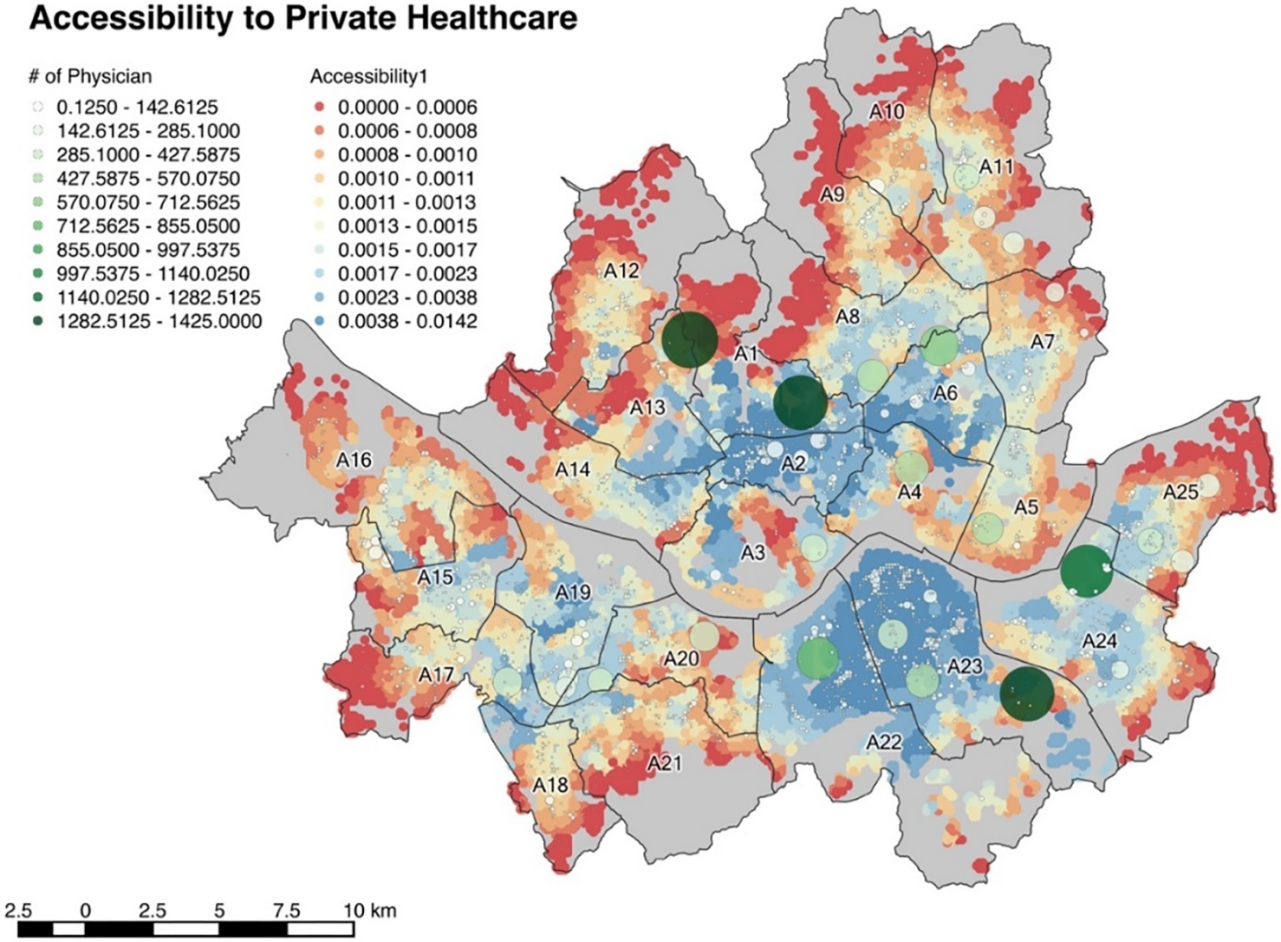
**Accessibility to private healthcare measured by (a) 2SFCA, (b) E2SFCA, (c) SE2FCA**.

Fig 2 shows the accessibility to private healthcare facilities in Seoul measured by three methods, 2SFCA, E2SFCA and SE2SFCA. Each dot represents the population grid with associated accessibility. When the color of a dot is closer to red, it represents lower accessibility while blue represents higher accessibility. The hospital grid, which overlaps the population grid, is represented by white and green circles that are spaced with 100 meters of distance. Greener and larger circles represent larger hospitals, while the smaller circles with whiter colors represent smaller hospitals.

The results from the three methods show differences in regional distribution. According to the results obtained from 2SFCA shown in Fig 2(A), the regions with high accessibility are mainly A3 and A4, followed by A5, A23, and part of A24. Overall, the accessibility to large hospitals has increased, which is the most prominent feature compared to the results obtained with the 2SFCA. In the vicinity of large hospitals located in A1, the accessibility varies according to the catchment area boundaries. This is due to the same catchment size regardless of the size of the hospital. The accessibility of nearby large hospitals is overestimated because they cannot grasp the service range of hospitals. The results obtained through SE2SFCA are different from the other two results. Unlike the results from the conventional methods, which shows a proximity between the higher accessibility areas, they are spread throughout Seoul. The problem of relatively high accessibility around the large hospitals has also been partially alleviated.

From the results of the accessibilities analysis with the proposed method, on average, 1.7843 doctors serve 1000 people in the city of Seoul. Considering the fact that the ratio of total demand and supplies is 2.53, the effective 1.7843 doctors are significantly lower than the total ratio. This result implies an unequal distribution on accessibility with large deviation within the city. The districts with higher accessibilities include A23, A22, A2, and A6. In these districts, there are many regular-sized healthcare complexes and private clinics. Suburban districts, including A12, A9, A10, and A11 are found to have generally lower accessibilities.

**Fig 3:**
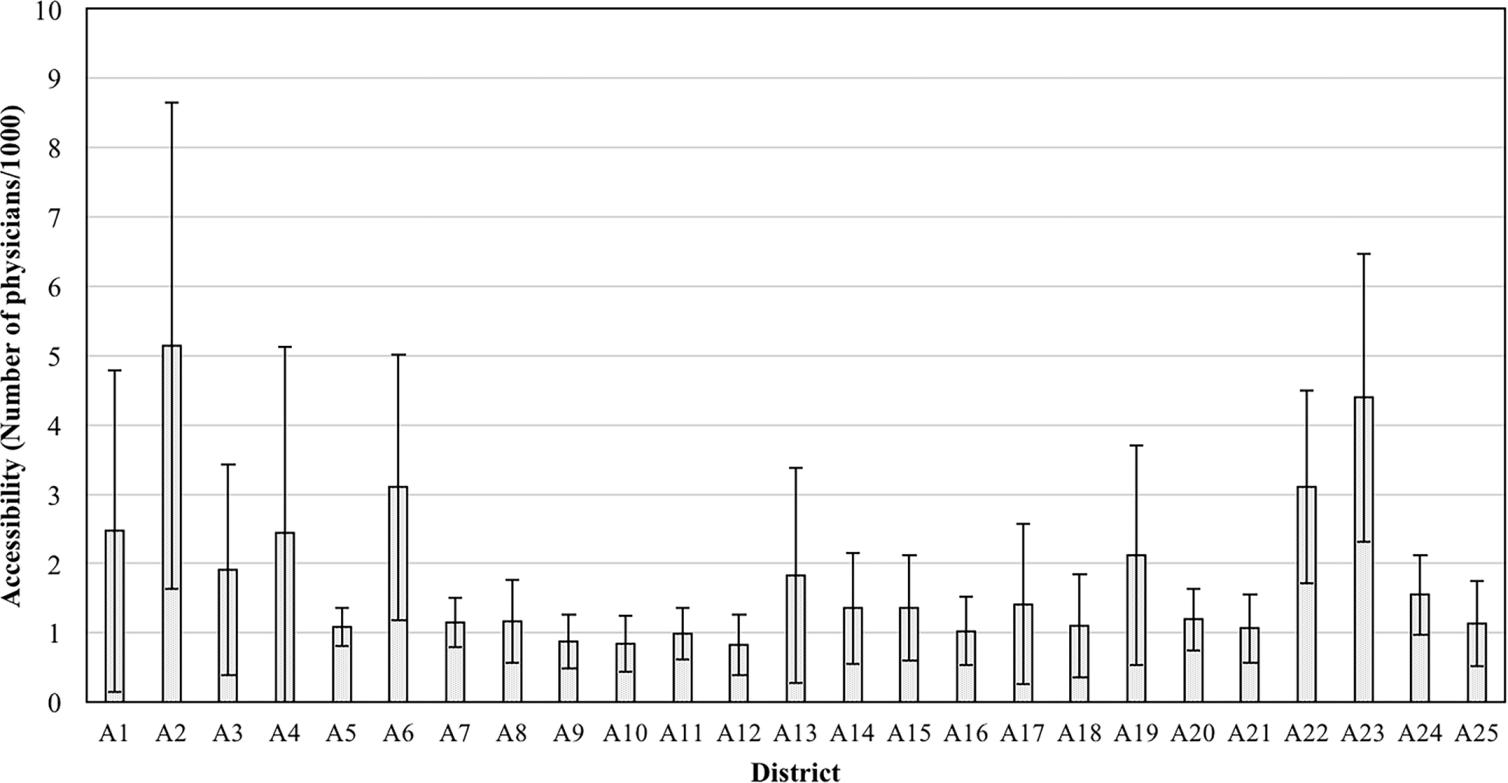
Average accessibility to private healthcare of districts.

The district with the highest accessibility is A2 with 5.1381 and A12 represents the lowest accessibility region with 0.8250. This value is roughly six times lower than A2. When the MBs within the districts are individually compared, the highest accessibility of 11.7661 is found to be A2, while A8 has the lowest accessibility with 0.1655. This value is approximately 70 times lower than A2. The results clearly show a great inequity in terms of healthcare accessibility distributions among the districts and MBs. Fig 3 shows the average accessibility among the districts of Seoul, and their associated numerical values are shown in Table 3.

**Table 3:** Accessibility to private healthcare (number of physician/1000).

District	Min	Max	Avg.	Std.
A1	0.4444	7.9733	2.4698	2.3245
A2	1.7505	11.7661	5.1381	3.5055
A3	0.4430	6.3977	1.9133	1.5166
A4	0.7255	11.5306	2.4476	2.6715
A5	0.6601	1.4909	1.0823	0.2712
A6	0.8245	9.3675	3.0982	1.9207
A7	0.7668	1.8454	1.1478	0.3561
A8	0.1655	2.3079	1.1682	0.5918
A9	0.2803	1.5434	0.8813	0.3894
A10	0.2994	1.6236	0.8393	0.4041
A11	0.4044	2.0023	0.9857	0.3726
A12	0.1696	2.5366	0.8250	0.4413
A13	0.5152	5.9550	1.8306	1.5510
A14	0.4069	3.9077	1.3544	0.8036
A15	0.5914	3.9003	1.3555	0.7584
A16	0.4781	2.8345	1.0264	0.4956
A17	0.3804	5.6440	1.4160	1.1544
A18	0.3449	2.7439	1.1024	0.7409
A19	0.9591	7.0088	2.1182	1.5885
A20	0.5635	1.9146	1.1919	0.4474
A21	0.3054	2.6010	1.0659	0.4902
A22	1.1441	5.0464	3.1030	1.3867
A23	0.8627	9.0337	4.3930	2.0789
A24	0.5547	2.5220	1.5478	0.5778
A25	0.2832	2.6978	1.1341	0.6165
Total	0.1655	11.7661	1.7843	1.6870

**Fig 4:**
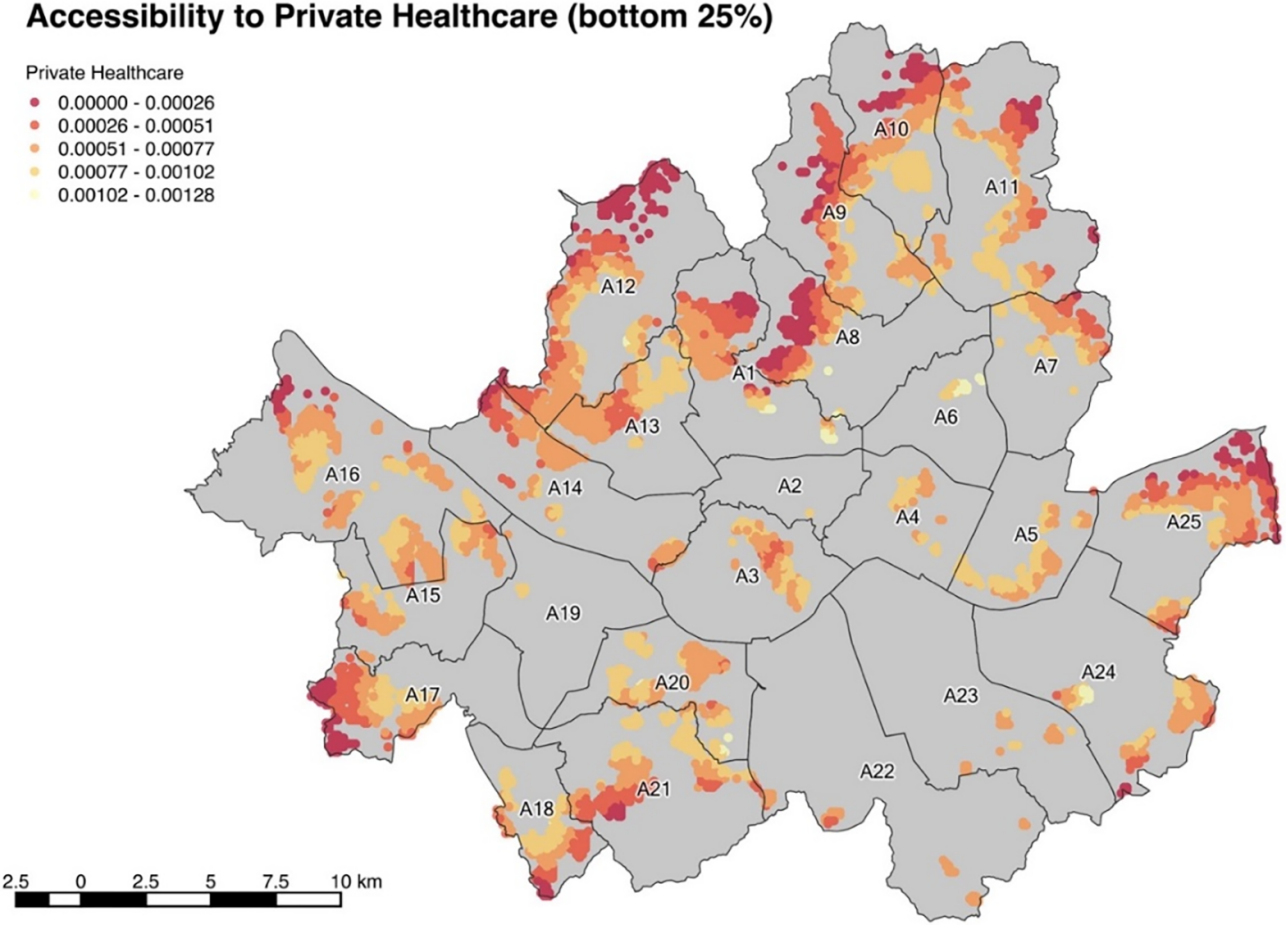
Accessibility to private healthcare (bottom 25%).

77.9% of the total population of Seoul have a lower accessibility than the average of 1.7843. The top 20% group experiences an above-the-average accessibility of 5.8026 while the bottom 25% group suffers from an accessibility of 0.5653. This shows how healthcare services are heavily biased towards the wealthier and higher social status-geographical groups. According to Fig 4, the central part and the South East part of Seoul has nearly no population that belongs to the bottom 25% group. A12 has the largest population belonging to the bottom 25% and A16, A11, A10, A21, A25, and A9 each has more than 150,000 people in the bottom 25% group. The population of the bottom 25% of each district is shown in Table 4.

**Table 4:** Population of bottom 25% accessibility to private healthcare.

Districts	Population	Districts	Population	Districts	Population
A1	38,718	A10	187,633	A19	2,936
A2	3	A11	180,554	A20	115,425
A3	59,562	A12	273,717	A21	173,849
A4	24,051	A13	137,613	A22	1,800
A5	106,554	A14	104,821	A23	13,919
A6	4,658	A15	102,827	A24	115,785
A7	95,345	A16	273,426	A25	168,513
A8	79,838	A17	123,619		
A9	152,396	A18	116,042	Total	2,653,604

Internationally unique aspects in terms of healthcare services in Seoul are the existences of highly concentrated mega-size hospital complexes. Four of such complexes are located in Seoul as shown in Fig 2 with large dark green circles. Each of them has more than 1000 healthcare staffs and roughly 200 times more physicians than the average of all hospitals in Seoul. Their service coverage nearly coincides with the city boundaries itself or even passes over to include other territories of nearby cities. The demand for those hospitals can reach beyond the boundaries of the city of Seoul due to the patients who prefer to be cared by such large hospitals with the assumption that large complexes would provide services that are more reliable. This is the reason why the resulting service supplies (R_j_) are not expected to be much larger (i.e. 200 times) than the other smaller private hospitals. The average Rj for those hospitals is 0.1795 and this value is roughly only 7 times of the average R_j_ for all hospitals of 0.0253, as the proposed method determines the coverage based on the size of the hospital.

The scope of this paper is geographically limited to the City of Seoul. Yet, these mega-size complexes also provide services to the regions outside of Seoul, which results in a lower effective service supplies (R_j_) to the City of Seoul. Due to this reason, the vicinities of the mega-complexes surprisingly do not benefit an increased accessibility.

A regression analysis is conducted in order to identify the factors that significantly influence the healthcare accessibility as shown in Table 5. The dependent variables are set as the accessibilities of each MB. The independent variables for each MB include average car ownership per person, average waiting time for public transportation, total healthcare employees, population, and the average IDI of MB. β means the magnitude of the relative influence of independent variables on dependent variables, and the result that shows the most significant factor with a positive correlation is the average car ownership. The average waiting time for public transportation and the number of population have a significant negative correlation with the accessibility. In step 2, multiple travel modes were considered and the modes depended largely on the vehicle ownership status. Since the critical travel distance varies in this step, it is expected that the two variables related to the population’s travel mode significantly influence the healthcare accessibility.

**Table 5:** Result of linear regression.

Dependent variable	independent variable	B	β	t	p
Accessibility	(constant)	.003			
	Avg. car ownership per person	.005	.625	20.164	.000***
	Avg. waiting time for public transportation	.000	-.074	-2.614	.009**
	Total healthcare employees	5.383E-7	.034	1.212	.226
	Population	-7.916E-8	-.295	-9.030	.000***
	Avg. IDI	-5.252E-5	-.030	-1.009	.313

** p < .01

*** p < .001

However, the total healthcare employees and average IDI do not significantly influence the accessibility. The accessibility measurement is conducted at MB level and Rj is calculated considering competitions among the demands. In other words, larger size facilities tend to receive more demands. Also, taking into account that unaffordable cases are not properly captured in the data set, the total healthcare employees may not have a significant influence on the accessibility.

### 2. Accessibility to public hospitals

The accessibility measurements for public healthcare facilities are conducted for IDI of 6 and below. 88.8% of the total population of Seoul, which are 9,208,687 in population, corresponds to the IDI of 6 or below. A total of 47 public healthcare facilities is included in this analysis. There are at least 2 public facilities in each district of Seoul and there are 197 physicians homogeneously assigned to those facilities. On average, there are 0.021 physicians for 1000 citizens.

**Fig 5:**
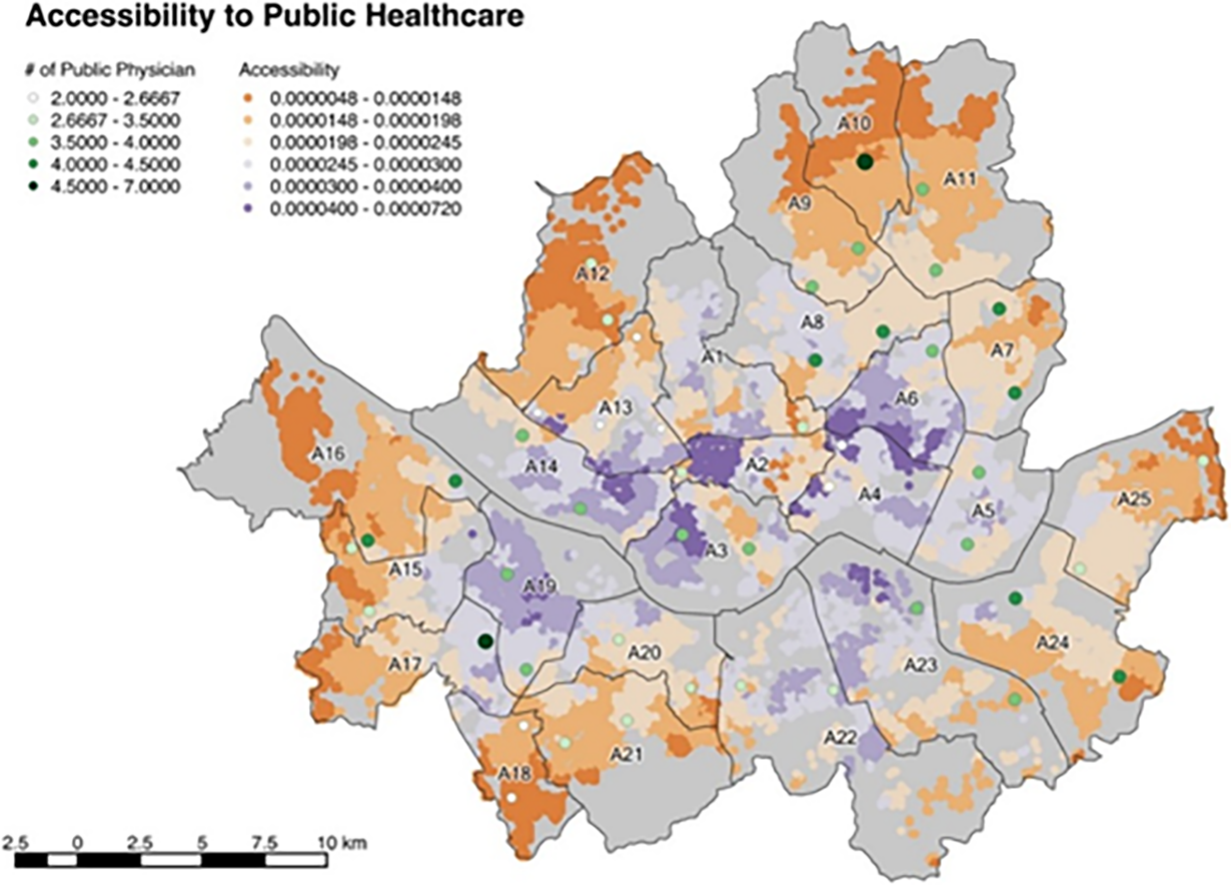
Accessibility to public healthcare.

Fig 5 shows the accessibility distribution in Seoul. The colors of the population grid illustrate the accessibility, where orange and purple colors represent low and high accessibilities respectively. It is noted that wealthy districts such as A23 and A22 are associated with a low accessibility to public hospitals compared with the accessibility to private healthcare. This is because these districts have small population with low income from the first place and its low-income population grids are inadequately associated with low accessibility.

On average, 0.0166 public hospital physician is assigned to every 1000 citizens in Seoul. The districts with higher public healthcare accessibilities are found to be A14, A19 and the central parts of Seoul. As in the case of the private hospitals, the outskirt districts have the lowest accessibility, which are A10, A12, A16, and A18.

**Fig 6:**
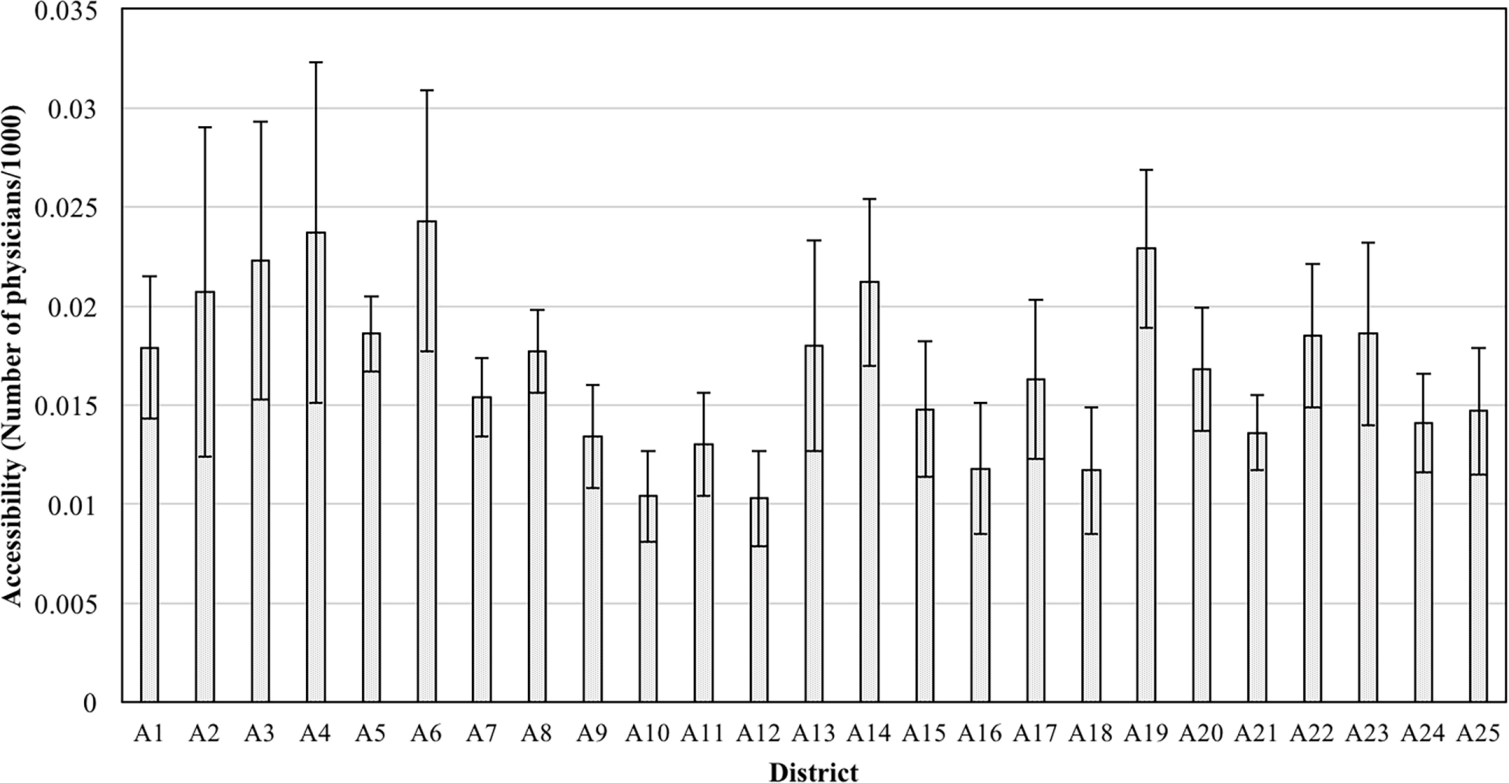
Average accessibility to public healthcare of districts.

A6 has the highest accessibility 0.0243 to the public healthcare and this value is approximately twice the lowest value of 0.0103. Fig 6 and Table 6 show the average accessibility of each district.

**Table 6:** Accessibility to public healthcare (number of physician/1000).

District	Min	Max	Avg.	Std.
A1	0.0085	0.0240	0.0179	0.0036
A2	0.0108	0.0360	0.0207	0.0083
A3	0.0138	0.0392	0.0223	0.0070
A4	0.0165	0.0375	0.0237	0.0086
A5	0.0155	0.0215	0.0186	0.0019
A6	0.0156	0.0436	0.0243	0.0066
A7	0.0125	0.0182	0.0154	0.0020
A8	0.0125	0.0217	0.0177	0.0021
A9	0.0093	0.0177	0.0134	0.0026
A10	0.0070	0.0133	0.0104	0.0023
A11	0.0089	0.0173	0.0130	0.0026
A12	0.0064	0.0184	0.0103	0.0024
A13	0.0122	0.0343	0.0180	0.0053
A14	0.0144	0.0330	0.0212	0.0042
A15	0.0098	0.0289	0.0148	0.0034
A16	0.0056	0.0177	0.0118	0.0033
A17	0.0111	0.0256	0.0163	0.0040
A18	0.0075	0.0194	0.0117	0.0032
A19	0.0156	0.0330	0.0229	0.0040
A20	0.0114	0.0230	0.0168	0.0031
A21	0.0109	0.0185	0.0136	0.0019
A22	0.0141	0.0238	0.0185	0.0036
A23	0.0119	0.0325	0.0186	0.0046
A24	0.0097	0.0207	0.0141	0.0025
A25	0.0081	0.0201	0.0147	0.0032
Total	0.0056	0.0436	0.0166	0.0057

**Fig 7:**
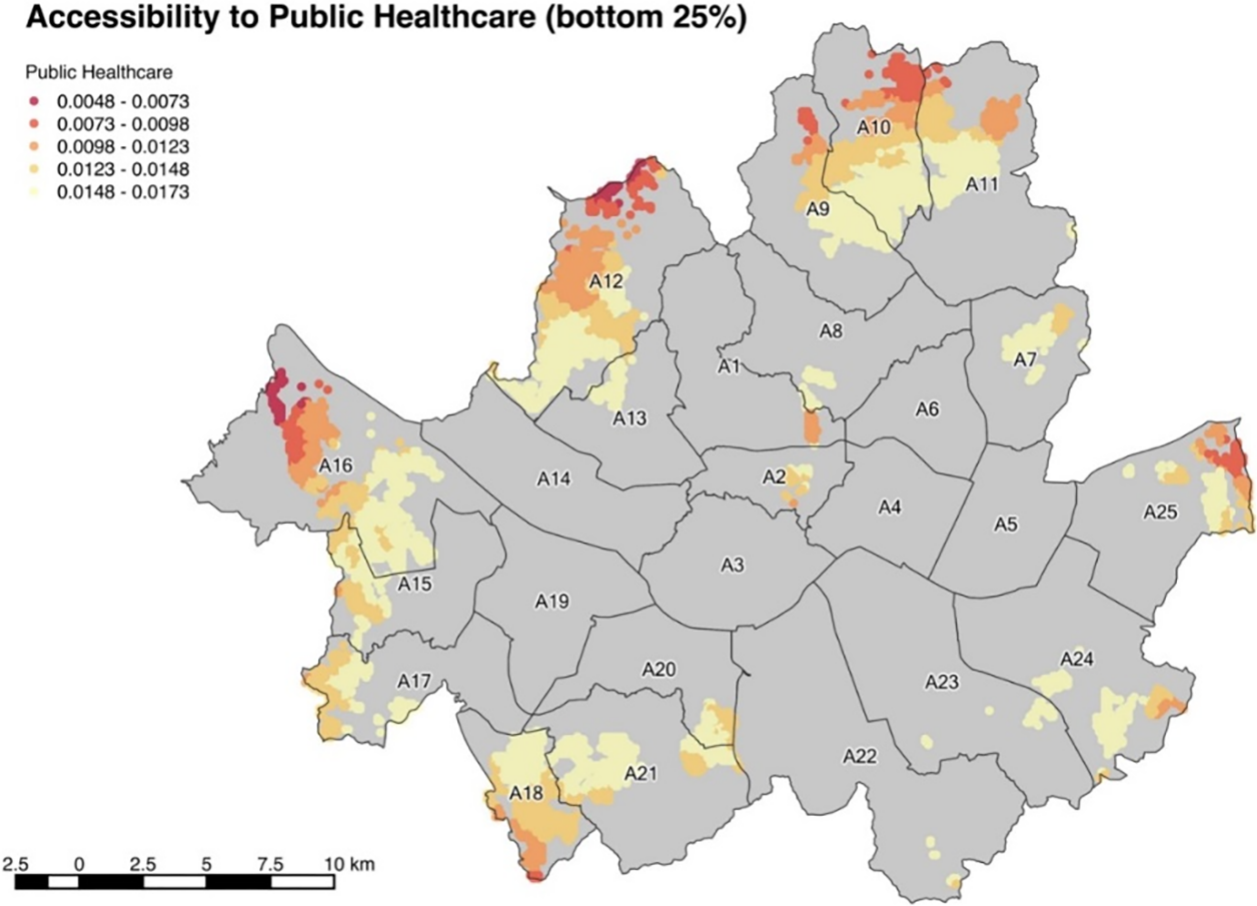
Accessibility to public healthcare (bottom 25%).

It is noted that the bottom 25% group of accessibility to public hospitals greatly overlaps with the associated group of accessibility to private hospitals. Fig 7 represents the bottom 25% area and the numerical values of the population included in the bottom 25% are shown in detail in Table 7.

**Table 7:** Population of bottom 25% accessibility to public healthcare.

Districts	Population	Districts	Population	Districts	Population
A1	9,788	A10	365,468	A19	-
A2	5,410	A11	274,877	A20	51,888
A3	-	A12	474,387	A21	192,377
A4	-	A13	18,447	A22	595
A5	-	A14	-	A23	20,828
A6	-	A15	115,008	A24	115,102
A7	70,428	A16	386,962	A25	62,359
A8	15,605	A17	55,483		
A9	131,694	A18	221,064	Total	2,587,769

In order to determine the factors that have a significant effect on public healthcare accessibility, a regression analysis is conducted as in Table 8. The dependent variables are set as the accessibility of each MB and the independent variables for each MB include the average car ownership per person, average waiting time for public transportation, population, and the average IDI of MB. The independent variables are consistent with the regression model for private hospitals, but the total healthcare employees are excluded from the variables because the hospital workforce is almost constant for public hospitals.

**Table 8:** Result of regression.

Dependent variable	independent variable	B	β	t	p
Accessibility	(constant)	3.407E-5			
	Avg. car ownership per person	1.380E-5	.452	11.848	.000***
	Avg. waiting time for public transportation	-1.221E-6	-.134	-3.850	.000***
	Population	-3.331E-10	-.348	-8.664	.000***
	Avg. IDI	-7.363E-7	-.116	-3.235	.001**

** p < .01

*** p < .001

As a result, the model shows that the most significant factor with a positive correlation is found to be the average car ownership with the highest absolute value of β. The average waiting time for public transportation, number of population, and average IDI are found to have negative correlations. Even if the result seems to be similar to the analysis of private healthcare accessibility, for the public healthcare, a relative importance of each factor is different. Since the deprived users of public healthcare have a narrower accessibility, the correlation of car ownership and accessibility is not as strong as in the case of private healthcare services. By contrast, the average waiting time for public transportation has a relatively significant impact because the low-income class with IDI of 6 and below generally tends to rely on the public transportation.

On the other hand, although the income class was not considered as a component to measure the accessibility, it has a meaningful impact on the accessibility of public healthcare. A result shows that the income class has a negative correlation with the accessibility. In other words, the lower income class tends to have a high accessibility to public healthcare. This is partially due to the fact that the government builds more public healthcare infrastructures in the regions with lower income levels.

### 3. Determination of vulnerable regions with low accessibilities

**Fig 8:**
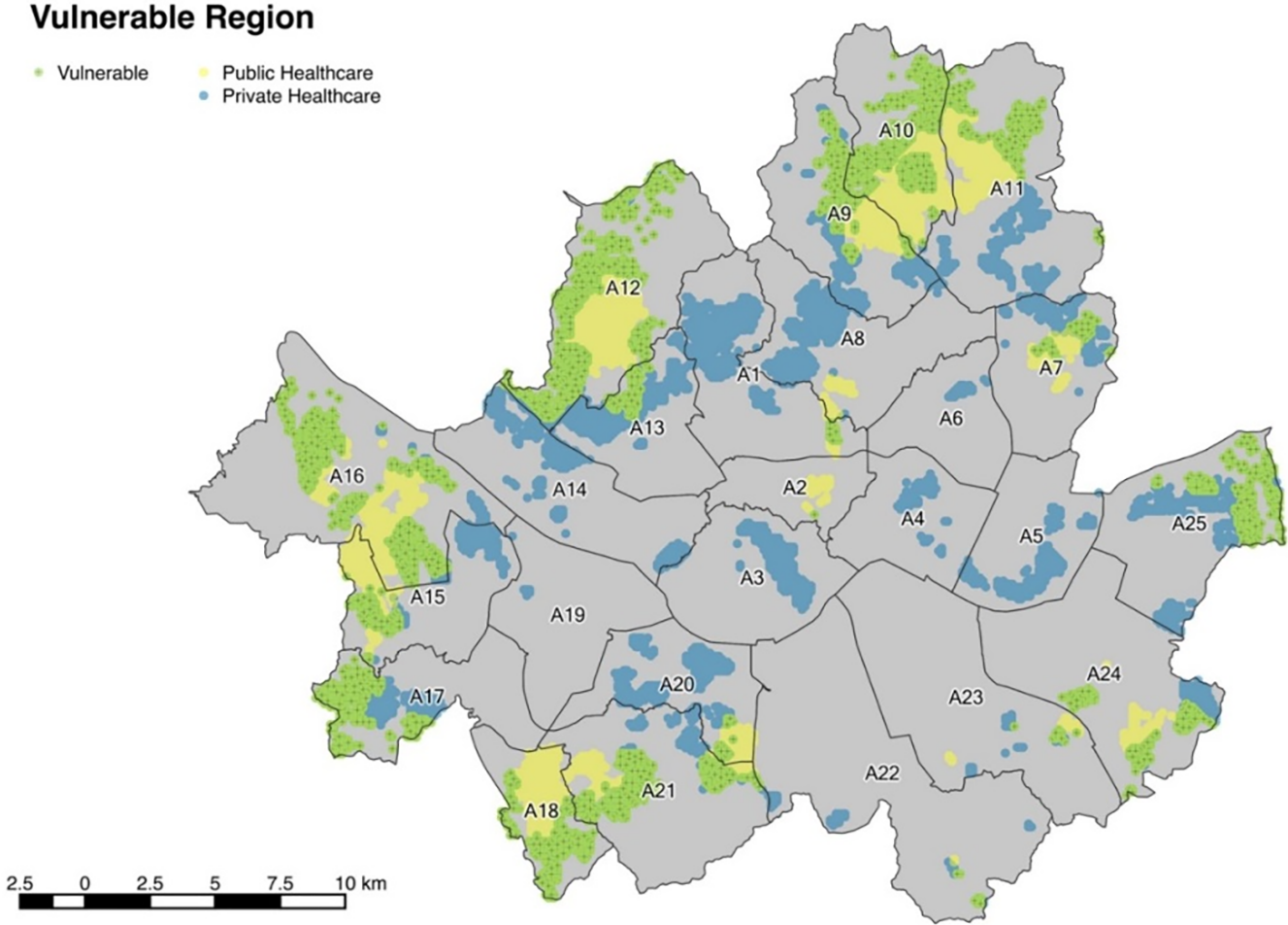
Healthcare weak accessibility region of Seoul.

This paper arbitrarily defines the bottom 25% of accessibility to be the threshold for problematic or weak accessibility regions. By spatially monitoring the bottom 25% regions for both private and public healthcare services, it is possible to identify the low accessibility regions. Fig 8 shows the weak accessibility regions. The blue color indicates the bottom 25% regions for private healthcare services while yellow color represents the bottom 25% regions for the public services. Light green regions are the common weak regions with the worst accessibilities in both private and public services.

Most of the weak regions are on the outskirts of Seoul and 13.2% of the population in Seoul are influenced. The average IDI is 3.76 for these regions and their average car ownership is only 0.2201 vehicles per person. For each healthcare-vulnerable district, Table 9 lists the population, average IDI, and average car ownership.

**Table 9:** Characteristics of weak accessibility region.

District	Population of vulnerable region	Avg. IDI	Avg. car ownership per person
A1	7,617	2.21	0.1557
A2	3	4.00	0.2376
A3	-	-	-
A4	-	-	-
A5	-	-	-
A6	-	-	-
A7	16,412	3.73	0.1907
A8	-	-	-
A9	48,501	3.52	0.1824
A10	183,072	3.50	0.1978
A11	73,472	3.70	0.2163
A12	257,046	3.59	0.2084
A13	18,440	4.09	0.1554
A14	-	-	-
A15	49,010	3.39	0.1904
A16	249,797	3.93	0.2464
A17	55,482	3.64	0.2796
A18	116,042	3.69	0.2222
A19	-	-	-
A20	13,026	3.67	0.1993
A21	132,092	3.86	0.2350
A22	528	5.80	0.5392
A23	6,585	5.08	0.2271
A24	80,489	4.67	0.1812
A25	62,359	4.23	0.2443
Total	1,369,970	3.76	0.2201

### 4. Qualitative review of results

This paper measures the healthcare accessibility to private and public hospitals in the City of Seoul, Korea and determines the regions with weak accessibility. The accessibility for private and public hospitals are found to share similar trends. The central parts of Seoul have high accessibility due to the establishment of tailored policies for the local needs. In all other parts of Seoul with an exception of South East parts of Seoul, the accessibility is significantly lower. However, those weak accessibility regions are still not generally considered for the accessibility enhancement in the municipal government presently. Based on the findings, the following recommendations are made for the decision and policy makers of the Seoul City.

Regarding the private healthcare accessibility, Seoul is currently experiencing a high degree of inequity in distributions. The accessibility is high for the central and South Eastern parts of Seoul due to the high car ownership and convenient public transit connections and schedules. However, other parts of Seoul, especially the outskirt districts are associated with low densities of healthcare infrastructures and relatively inconvenient public transit services. One possible proactive solution to remedy the inequity in accessibility is to enhance the public transit systems in the weaker regions. By strategically building new transit routes with optimally located stops, and implementing improved bus frequency, it is possible to enhance the existing transit system in a way that would increase the healthcare accessibility. This would directly improve the usage of the public transit system, which in turn would increase the accessibility by reducing the travel time to the hospital networks. The enhancement in public transit would also induce new hospitals to open in the weaker regions.

According to the regression analysis that shows a negative relationship between the accessibility and income level, public hospitals are being serviceable to a certain degree. However, the outskirt regions are in desperate needs of improvement. In particular, these areas require strong public services as they are generally less accessible to private hospitals.

For regions with a low accessibility to both private and public healthcare services, visiting or remoting healthcare programs need to be implemented or enhanced further to remedy the severity of the inequity in accessibility. Even if such services are being discussed and designed in the city’s administrations, they are not yet implemented. The results from this paper can help determine the regions to include for the improvement.

## Conclusion

This paper recognizes that current existing healthcare accessibility measurement methods are inadequate for the City of Seoul, Korea. Therefore, we developed a noble approach that considers various factors that are unique to Seoul City. The proposed SE2SFCA method is applied to private and public healthcare infrastructures in Seoul for determining districts and MBs with low healthcare accessibilities.

The proposed SE2SFCA resolves the issue of over-evaluating the accessibility for regions near larger hospitals. By incorporating probabilistic approachability to certain locations, the method is found to effectively eliminate the over-evaluation of accessibility near regions with a high population and hospital density. (i.e. service coverage areas of mega-size healthcare complexes are so large that they do not necessarily contribute to serving the regions in the vicinity)

By conducting the healthcare accessibility analysis for both private and public hospitals, the vulnerable regions with low accessibility have been investigated and the relative recommendations have been made. The vulnerable districts are found to be A10, A12, A16, A18, and A21, which are mostly the outskirts of the city. The proposed method is expected to be applicable to other cities in Korea and to other international cities that share similar attributes with Seoul in terms of population size and hospital density in a relatively small geographical area including Hong Kong, Beijing, Tokyo, and etc.
