# Correction: Enhancing healthcare accessibility measurements using GIS: A case study in Seoul, Korea

**DOI:** 10.1371/journal.pone.0194849

**Published:** 2018-03-20

**Authors:** Yeeun Kim, Young-Ji Byon, Hwasoo Yeo

Fig 2 is missing panels B and C. Please see the corrected Fig 2 here.

There is an error in the caption for Fig 2. Please see the correct Fig 2 caption here.

**Fig 2:**
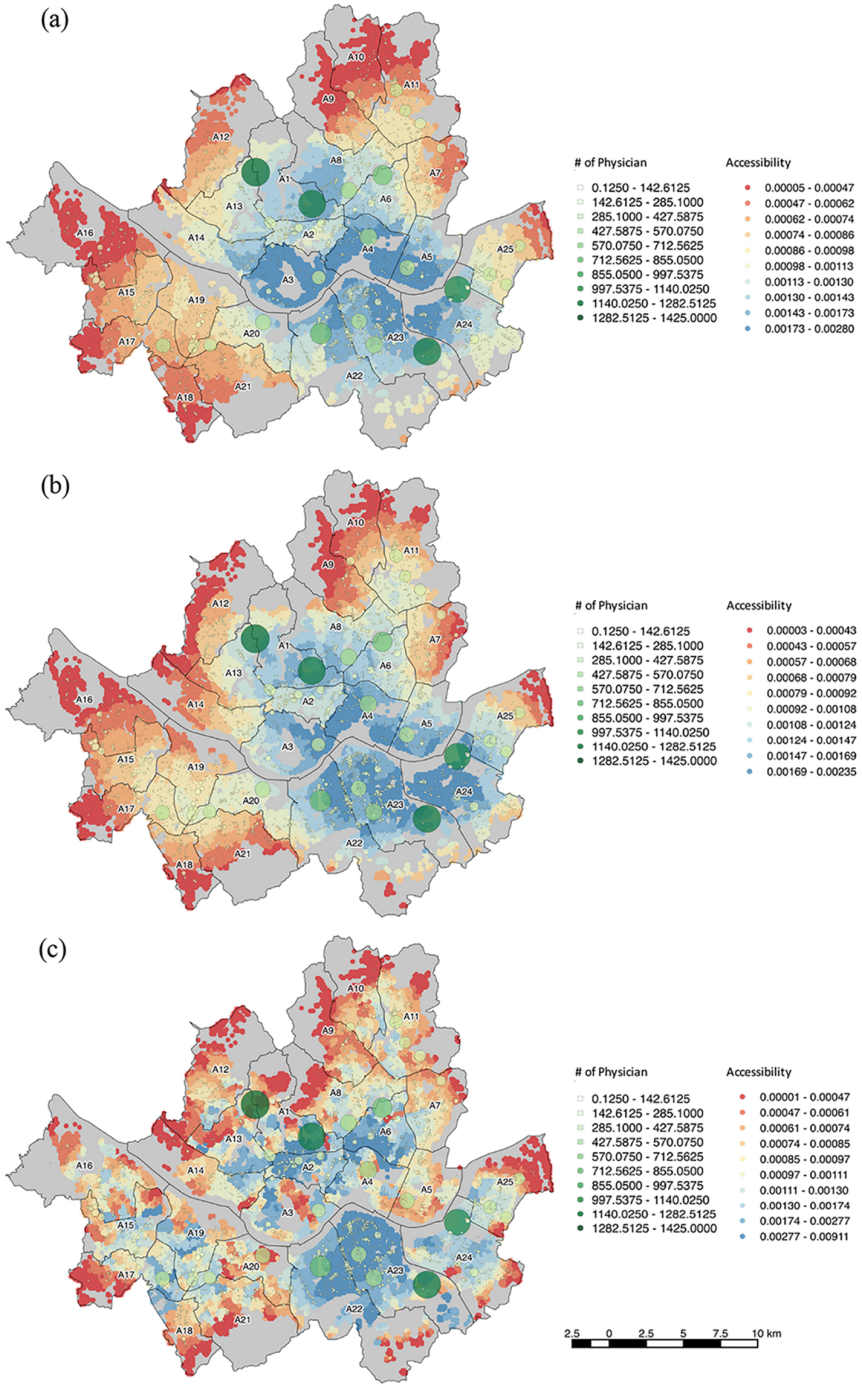
Accessibility to private healthcare measured by (a) 2SFCA, (b) E2SFCA, (c) SE2SFCA.
